# Supplementary material for: Epigenomic heterogeneity and spatial organization during human reprogramming
Source: bioRxiv. 2026 Oct 21:2026.10.15.757424. Preprint. [Version 1] doi: 10.64898/2026.10.15.757424 (PMC13738419; doi:10.64898/2026.10.15.757424)
Supplement: Supplementary file 1 [file NIHPP2026.10.15.757424v1-supplement-2.pdf]

bioRxiv preprint doi: https://doi.org/10.64898/2026.10.15.757424; this version posted October 21, 2026. The copyright holder for this preprint (which was not certified by peer review) is the author/funder, who has granted bioRxiv a license to display the preprint in perpetuity. It is made available under a CC-BY 4.0 International license.

Supplemental Notes

Supplementary Note 1

On average, 10x Multiome nuclei contained 7,500 RNA UMIs and 21,000 ATAC fragments (**Extended Data Figures 1D-E**). On average, snmCT-seq profiles detected 80,000 read fragments per nucleus (**Extended Data Figure 1F**) and had a mean methylome coverage of 2.5% of the human genome (**Extended Data Figure 1G**). The snm3C-seq profiles had a mean methylome coverage of 2.8% of the human genome and contained approximately 81,000 long-range intrachromosomal interactions spanning ≥1 kb per nucleus **(Extended Data Figures 1H-I).** The mean intrachromosomal-to-interchromosomal interaction ratio was approximately 2.0, within the range of similar snm3C-seq experiments[34] (**Extended Data Figure 1J**). Because of insufficient cell numbers, C37 and C38 were not profiled at days 14–18 by snmCT-seq or at days 16–18 by snm3C-seq. All of the reported QC metrics per-nucleus can be found in **Table S2.**

Supplementary Note 2

We used shared DNA methylation measurements to transfer RNA-defined cell-state labels from snmCT-seq to snm3C-seq. To evaluate this approach, we tested how accurately methylation predicted cell states within snmCT-seq, where RNA and methylation were measured in the same nuclei. The classifier achieved a mean F1-score of 0.73 on held-out nuclei, but poorly resolved Skin and Inflamed cell populations (**Extended Data Figure 3I**). Modifications to the classifier, including increasing the representation of underrepresented cell states in the training data and testing alternative methylation feature sets, had limited effect on the classification (**Extended Data Figures 3J–K**). We also compared methylation profiles between snmCT-seq and snm3C-seq, finding similar global methylation distributions (**Extended Data Figure 3G**). To assess agreement at the cell-state level after label transfer, we identified differentially methylated regions (DMRs) between cell states in snmCT-seq and compared methylation in the corresponding annotated states in snm3C-seq (excluding Skin/Inflamed due to low cell number). State-level methylation patterns across these DMRs were similar between assays (**Extended Data Figure 3L**), as were methylation-based trajectory-score estimates (**Extended Data Figure 3M**).

Supplementary Note 3

We used two complementary approaches to characterize reprogramming states in the spatial data: a CosMx-specific trajectory score and transfer of RNA-defined cell-state labels from the 10x Multiome. First, we derived a fibroblast-to-iPSC trajectory score based on a 1K CosMx reference dataset of 8,775 pre-transduction fibroblasts and 30,265 mature-iPSCs (at passage 17 after derivation from the reprogramming culture). These reference samples provided starting and mature-iPSC endpoints, which were not sampled in the primary 6K CosMx time course spanning days 3-13. We applied this score to individual cells in the primary 6K CosMx dataset, providing a continuous measure of fibroblast-to-iPSC transcriptional progression to complement the transferred cell-state annotations (**Figure 7B and Extended Data Figures 11B,C**).

Second, to transfer cell-state labels, we extended our XGBoost-based classification scheme (**Figure 1B**) to account for differences between whole-cell CosMx and single-nucleus

bioRxiv preprint doi: https://doi.org/10.64898/2026.10.15.757424; this version posted October 21, 2026. The copyright holder for this preprint (which was not certified by peer review) is the author/funder, who has granted bioRxiv a license to display the preprint in perpetuity. It is made available under a CC-BY 4.0 International license.

10x Multiome measurements. We initially noted systematic misclassification of day 3 OSKM-high cells (putative "Sendai" state) as "Inter2" and "iPSC." We hypothesized that greater detection of cytoplasmic Sendai-derived OSKM transcripts in whole-cell CosMx measurements than in the 10x Multiome single-nucleus RNA profiles contributed to this misclassification. We excluded the four OSKM genes, sex chromosome genes, and 365 genes with large between-platform effects before XGBoost model training (see Methods).

The final classifier achieved a multiclass F1-score of 0.91 (mean across cell states, held-out 10x reference data). The assigned states showed the expected CosMx-derived trajectory scores (**Extended Data Figure 11C**). Marker gene expression also supported these annotations (**Extended Data Figure 11D**), and state-level expression patterns were concordant between CosMx and the 10x Multiome (**Extended Data Figure 11E**). Genes with large between-platform differences included those encoding endoplasmic reticulum (ER)-associated, membrane and secreted proteins (e.g., *APOE*, *CYP1B1*, *TMEM119*). These differences may reflect subcellular RNA distribution[73] and assay-specific differences in transcript detection.

Supplementary Note 4

We observed substantial differences in cell-state proportions between CosMx and 10x Multiome profiling. Compared with dissociation-based 10x Multiome profiling, the CosMx spatial datasets contained lower proportions of Inter2 and early-iPSC cells and higher proportions of Fail1 and Fail2 cells at days 9 and 13 (**Extended Data Figures 11F,G**). The low representation of pluripotency-associated cells in the spatial data is qualitatively consistent with the sparse NANOG-positive clusters detected by independent imaging (**Extended Data Figures 12H,I**). The spatial data also showed a higher proportion of early-iPSC cells in C29 than in C38 (**Extended Data Figure 11F**), qualitatively consistent with the donor differences in independent *NANOG*-positive colony counts detected using antibody immunofluorescence staining (**Extended Data Figure 12I**). C29 also contributed more nuclei than C38 to the early-iPSC population in the 10x dataset (**Extended Data Figure 7A**). Collectively, the higher representation of early-iPSC nuclei in the dissociation-based datasets could reflect preferential recovery of early-iPSC cells or their nuclei during sample preparation, whereas spatial profiling measures cell-state composition within the intact culture.

Supplementary Note 5

We used MISTy[74] to estimate contributions to gene-expression predictions from the expression of other genes within the same cell, immediate neighbors (in MISTy terminology, juxtacrine-scale; ≤30 μm) and a broader, distance-weighted neighborhood (paracrine-scale; Gaussian-weighted based on distance to cell, up to 750 μm) (**Table S13**). We assessed overall model performance (r^2_{tot}) using the MISTy "meta-model" pooled from all three views. Estimating the predictive contributions assigned to each spatial scale, we found that the cell's own profile (r^2_{intra}) made the largest contribution for most genes, whereas contributions from immediate neighbors (r^2_{juxta}) and the broader neighborhood (r^2_{para}) were smaller on average. However, the relative contribution from immediate neighbors varied among genes and was comparatively large for pluripotency- and chromatin-associated genes, including *NANOG*, *DPPA3*, and *DNMT3L*, as well as the cell-cycle

bioRxiv preprint doi: https://doi.org/10.64898/2026.10.15.757424; this version posted October 21, 2026. The copyright holder for this preprint (which was not certified by peer review) is the author/funder, who has granted bioRxiv a license to display the preprint in perpetuity. It is made available under a CC-BY 4.0 International license.

gene *PLK1*. Relatively large broader-neighborhood contributions were observed for *SFRP2*, *IGFBP5*, and *SPP1* when averaged across time points (**Extended Data Figure 15A**).

The neighborhood-associated contributions were correlated between the primary dataset and the independent 1K CosMx experiment (MISTy run on top 500 highly variable genes, same settings otherwise). Subsetting to the intersection of genes modeled in both datasets, we observed Spearman's ρ = 0.80 for $r^2_{niche} = (r^2_{juxta} + r^2_{para})/r^2_{tot}$ between the primary 6K and independent 1K CosMx datasets. Further, the set of 50 genes with the highest neighborhood-associated contributions based on r^2_{niche} shared 37 genes, compared with the 15 expected by chance (2.5-fold enrichment; hypergeometric $P = 1.5\times10^{-15}$).

We separately examined which genes were more important predictors of other genes by counting how frequently each gene ranked among the top 50 predictors based on random forest variable importance scores. Gene-set analysis of broader-neighborhood predictor rankings identified enrichment for coagulation (ECM-remodeling), interferon-α and interferon-γ responses, MYC targets and oxidative phosphorylation (**Extended Data Figure 15B**, Hallmark gene sets FDR < 0.10). These predictive associations identify spatial covariance; they do not establish signaling or the direction of regulation. Together, these analyses identify gene-specific patterns of transcriptional covariation within cells and across immediate and broader neighborhoods during reprogramming.

bioRxiv preprint doi: https://doi.org/10.64898/2026.10.15.757424; this version posted October 21, 2026. The copyright holder for this preprint (which was not certified by peer review) is the author/funder, who has granted bioRxiv a license to display the preprint in perpetuity. It is made available under a CC-BY 4.0 International license.

Supplementary Table Summary

Table S1: Cell line information

Table S2: Single nucleus metadata with QC columns

Table S3: Methylation-based ChromHMM states and enrichments with external datasets

Table S4: Reference PMD set identified in fibroblasts, snmCT-seq

Table S5: Differential-expression test results from 10x Multiome

Table S6: Differentially open chromatin regions identified in 10x Multiome

Table S7: ATAC-based ChromHMM states and enrichments with external datasets

Table S8: Trimodal quantification of subcluster marker features

Table S9: Autosomal chromatin compartment assignments by cell state

Table S10: Gene bin assignments based on timing in productive reprogramming

Table S11: Radial gene programs, including per-cluster membership and gene metadata

Table S12: Sparse Canonical Correlation Analysis networks between gene expression, morphology, and spatial variables in the spatial data

Table S13: MISTy model performance, model-assigned predictive contributions, predictor importance scores and gene set enrichments

Table S14: Significant ligand-receptor interactions detected using physical-distance aware CellChat
